# Competing contagion processes: Complex contagion triggered by simple contagion

**DOI:** 10.1038/s41598-018-28615-3

**Published:** 2018-07-10

**Authors:** Byungjoon Min, Maxi San Miguel

**Affiliations:** 1 IFISC, Instituto de Física Interdisciplinar y Sistemas Complejos (CSIC-UIB), Campus Universitat Illes Balears, E-07122 Palma de Mallorca, Spain; 2 Department of Physics, Chungbuk National University, Cheongju, Chungbuk 28644 Korea

## Abstract

Empirical evidence reveals that contagion processes often occur with competition of simple and complex contagion, meaning that while some agents follow simple contagion, others follow complex contagion. Simple contagion refers to spreading processes induced by a single exposure to a contagious entity while complex contagion demands multiple exposures for transmission. Inspired by this observation, we propose a model of contagion dynamics with a transmission probability that initiates a process of complex contagion. With this probability nodes subject to simple contagion get adopted and trigger a process of complex contagion. We obtain a phase diagram in the parameter space of the transmission probability and the fraction of nodes subject to complex contagion. Our contagion model exhibits a rich variety of phase transitions such as continuous, discontinuous, and hybrid phase transitions, criticality, tricriticality, and double transitions. In particular, we find a double phase transition showing a continuous transition and a following discontinuous transition in the density of adopted nodes with respect to the transmission probability. We show that the double transition occurs with an intermediate phase in which nodes following simple contagion become adopted but nodes with complex contagion remain susceptible.

## Introduction

Models of social and biological contagion in general fall into two classes depending on the response to successive exposures: simple and complex contagion^1–12^. Simple contagion, mainly inspired by disease spreading, stands for a contagion process with independent interaction between the susceptible and the infectious^5,7,13–19^. Typical compartment epidemic models such as the susceptible-infected-recovered model^5,13,15,16^ and the susceptible-infected-susceptible^5,14,16^ model belong to the class of simple contagion processes. Models of simple contagion are controlled by an infection probability independent of the number of exposures. Typically, a model of simple contagion exhibits a continuous phase transition between an epidemic phase and a disease free phase for a critical value of the infection probability. The other class of contagion processes is complex contagion representing spreading phenomena in which multiple exposures to a spreading entity are needed for changing agents’ state^8,9^. Models of complex contagion processes encompass a wide range of contagious models such as threshold model^4,6,20^, generalized epidemic model^21–25^, diffusion percolation^26^, threshold learning^27,28^, and bootstrap percolation^29^. The spread of fads, ideas, and new technologies in our society is better described by complex contagion rather than by simple contagion due to a collective effect in social contagion^30–35^. The critical difference of the complex contagion as compared to the simple contagion processes is that the probability of adoption depends on the number of exposures. For instance, in the threshold model the adoption of a new innovation happens when the number of adopted neighbors is larger than a certain threshold^4,6^. Models of complex contagion often result in a discontinuous phase transition in contrast to the continuous phase transition of simple contagion^6,33,36–39^.

Classical contagion models assume that the contagious entity determines the type of contagion either simple or complex^16,30^. Recently, the comprehensive analysis of the spread of an equal-sign profile in a social networking service (SNS)^40–42^ sheds light on the mechanisms of contagion processes between the susceptible and the adopted. The analysis of the empirical data shows that characteristics of the agents also affects the type of contagion^41^. Specifically, it is observed that the number of successful exposures requiring for adoption is far different for different individuals^41^. In this observation, some agents change their profile in SNS just after the first exposure to the meme (simple contagion), but the others need more exposures to be adopted (complex contagion). This implies a competition between simple and complex contagion depending on agents’ adoptability, deviating from the traditional view of contagion models. The heterogeneity of adoptability can be widespread for many spreading phenomena because of the individual diversity of stubbornness, creed, and preexisting information. These facts call for incorporating such heterogeneity when modelling contagion processes integrating simple and complex contagion^43–47^. Incorporating such heterogeneity, here we propose a contagion model that in addition considers a transmission probability in the contagion process acting like an infection probability in epidemic models or an occupation probability in bond percolation processes on a network. It represents a trial of transmission from adopted neighbors, prior to the subsequent adoption processes. Effectively the transmission probability acts as a simple contagion process triggering a process of complex contagion.

In our model of contagion processes with a transmission probability we unify simple and complex contagion by considering agents with heterogeneous adoptability. We assign explicitly a different level of adoptability for individuals to mimic the heterogeneity of adoptability observed in empirical data^41^. The transmission probability models a chance to transmit and to identify successful (active) connections for adoption processes. With these generalizations, our model includes a variety of contagion models such as the susceptible-infected-recovered model^13^, threshold model^4,6^, diffusion percolation^26^, and bootstrap percolation^48^. Our generalized contagion model exhibits a rich variety of phenomena including continuous, discontinuous, and hybrid phase transitions, criticality, tricriticality, and double transitions. We show that a double transition with an intermediate phase can happen when a system is composed of nodes with heterogeneous adoptability.

## Generalized Contagion Model

We consider a network with *N* nodes that can be in a susceptible or adopted state. The adoptability *θ* of each node is randomly drawn from a distribution *Q*(*θ*). To be specific, *θ* represents the number of successful exposures required to change from susceptible to adopted. For example, when *θ* = 1, a node becomes adopted after a single successful exposure thus indicating simple contagion, while when *θ* > 1, it represents complex contagion node since multiple exposures are needed for adoption. Varying the adoptability *θ*, we can describe both simple and complex contagion processes. The chance of transmission is determined by a transmission probability *λ*. Each adopted node attempts to spread with the probability *λ* and hence we can identify active connections between the susceptible and the adopted. Introducing the distribution of adoptability *Q*(*θ*) and the transmission probability, we unify the two contagion mechanisms and suggest a generalized contagion model. It is worthwhile to note that heterogeneous adoptability but without a transmission probability was considered in the threshold model^4,42^, heterogeneous *k*-core percolation^45,46^, and a model of stochastic interacting particles^49^.

In our model, dynamics is in discrete time. Initially, all nodes are susceptible except a fraction *ρ* of seed nodes that are adopted. Newly adopted nodes attempt transmission with a probability *λ* to all of their susceptible neighbors in the same time step. In the next time step, each susceptible node updates the number of successful transmissions and becomes adopted if the number of successful exposures is the same or larger than its threshold. In more detail: let us suppose that an adopted node *i* tries transmission to its susceptible neighbor *j* with a probability *λ*. If the transmission is successful, the link from *i* to *j* becomes active and with the complementary probability 1 − *λ*, the link remains inactive. Then, susceptible node *j* becomes adopted when the number of successful exposures (equivalently the number of active links towards node *j*) exceeds or equates its adoptability *θ*_*j*_. This process proceeds until there are no more newly adopted agents in a network.

The main parameters of our model are *λ* and *Q*(*θ*) which reflect respectively the extent of transmissibility of a contagious entity and the adoptability distribution of the nodes. Depending on these two parameters, our model becomes one of a wide range of contagion models. The susceptible-infected-recovered model^13,15^ is recovered when (*λ*, *Q*(*θ*)) = (*λ*, *δ*_*θ*,1_) where *δ*_*i*,*j*_ represents the Kronecker delta function (the function is 1 if *i* = *j* and 0 otherwise). Diffusion percolation^26^ corresponds to (*λ*, *Q*(*θ*)) = (1, *δ*_*θ*,*n*>1_) where *n* is any integer larger than unity, while Watts’ threshold model^6^ corresponds to (*λ*, *Q*(*θ*)) = $(1,{\delta }_{\theta ,{k}_{i}T})$ where *T* is a threshold and *k*_*i*_ is the degree of node *i*.

**Figure 1:**
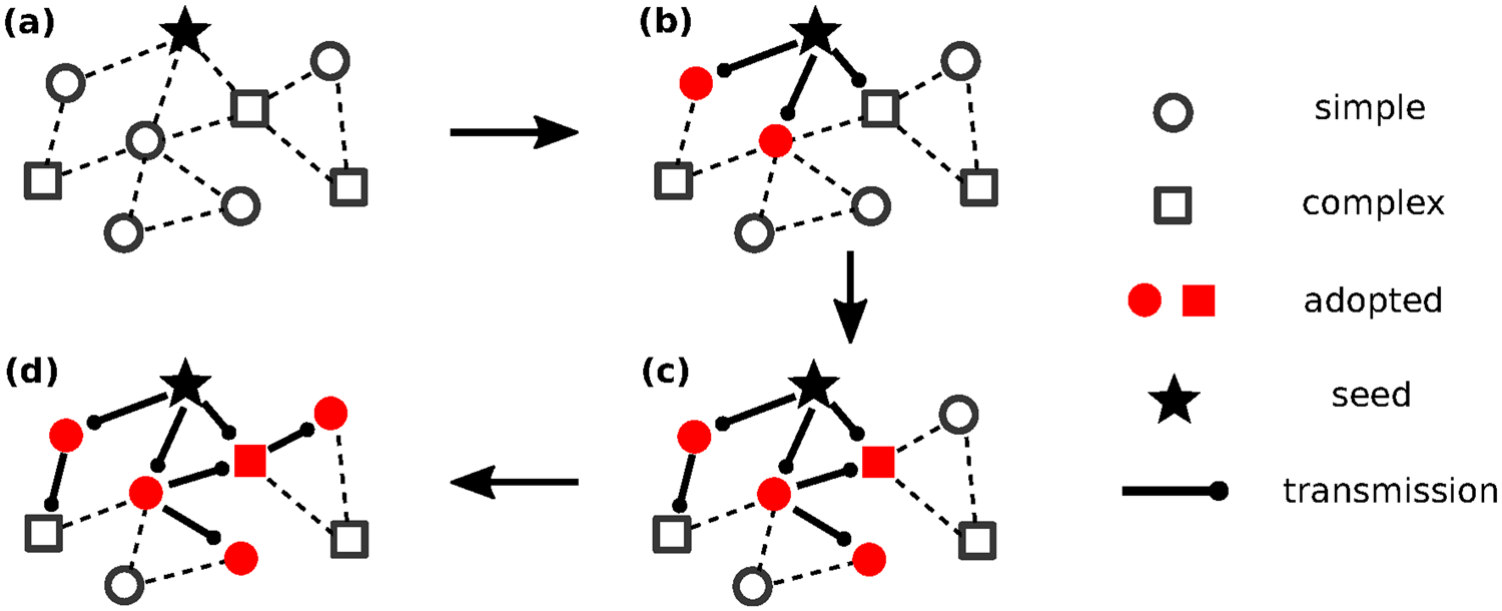
An example of our model of contagion processes with a transmission probability unifying simple and complex contagion. In this example, five nodes (circles) out of nine nodes follow simple contagion and three nodes (squares) follow complex contagion requiring two exposures to be adopted. Spreading starts from a seed (star symbol) and susceptible nodes (open symbols) become adopted (filled symbols) when the number of successful exposures exceeds or equates its assigned adoptability either 1 for simple contagion or 2 for complex contagion.

Figure 1 shows an example of our generalized contagion model with *Q*(*θ*) = (1 − *p*)*δ*_*θ*,1_ + *pδ*_*θ*,2_. A fraction (1 − *p*) of nodes denoted by circles follow simple contagion (*θ* = 1) and a fraction *p* of nodes denoted by squares follow complex contagion requiring multiple successful exposures to become adopted (*θ* > 1). Initially, all nodes are susceptible except a seed indicated with a star symbol [Fig. 1(a)]. Next, adopted nodes attempt to spread the contagious entity with a probability *λ*. If a trial is successful, a susceptible node is exposed to a contagious entity (denoted by thick line). Note that a single success of transmission does not always result in adoption because complex contagion requires multiple successful exposures. When the number of successful exposures exceeds or equates the adoptability *θ* of a node, a susceptible node turns to the adopted state (filled symbols) [Fig. 1(b–d)]. Eventually we measure the final fraction of adopted nodes *R* at the steady state.

## Analytical Approach

To predict the final fraction of adopted nodes, we derive mean-field equations assuming a locally tree-like structure in the limit *N* → ∞. Our approximation is exact in a tree structure and it gives very good agreement with numerical simulations for sparse random graphs with only infinite loops. Our approach is based on recent theoretical developments for the threshold cascade model on networks^36^. A generating function technique developed for a model of percolation processes^15^ also shares the idea of our analytical treatment. Given a degree distribution *P*(*k*) and an adoptability distribution *Q*(*θ*), the expected final fraction of adopted nodes *R* from a fraction of initial seed nodes *ρ* (chosen randomly) can be expressed as^15,36^ [see details in Supplementary Information],

1 $$R=\rho +\mathrm{(1}-\rho )\sum _{k=0}^{\infty }\,P(k)\sum _{m=0}^{k}\,(\begin{array}{c}k\\ m\end{array})\,{q}_{\infty }^{m}{(1-{q}_{\infty })}^{k-m}\sum _{\theta =1}^{\infty }\,Q(\theta )[1-\sum _{s=0}^{\theta -1}\,(\begin{array}{c}m\\ s\end{array})\,{\lambda }^{s}{(1-\lambda )}^{m-s}].$$

Here *q*_∞_ is the steady state probability that a node is adopted by following a randomly chosen link, and *λ* is the transmission probability between the susceptible and the adopted. The term $$(\begin{array}{c}k\\ m\end{array})\,{q}_{\infty }^{m}\,{(1-{q}_{\infty })}^{k-m}$$ corresponds to the probability of having *m* adopted neighbors out of *k* neighbors. And, $$[1-{\sum }_{s=0}^{\theta -1}\,(\begin{array}{c}m\\ s\end{array})\,{\lambda }^{s}{(1-\lambda )}^{m-s}]$$ represents the probability that the number of successful exposures with the transmission probability *λ* fro*m m* adopted neighbors exceeds or equates the adoptability *θ*. Overall, Eq. 1 corresponds to the probability that a randomly chosen node is either a seed node with probability *ρ* or is not a seed with the probability (1 *−* *ρ*) but it becomes eventually adopted in the dynamical process.

The probability *q*_∞_ can be obtained by solving a recursive equation. First we define *q*_*t*_ as the probability that a node is adopted by following a randomly chosen link at level *t*. On a locally tree-like graph, *q*_*t*_ can be obtained by (derivation in Supplementary Information)

2 $${q}_{t+1}=\rho +(1-\rho )\sum _{k=1}^{\infty }\,\frac{kP(k)}{\langle k\rangle }\sum _{m=1}^{k-1}\,(\begin{array}{c}k-1\\ m\end{array})\,{q}_{t}^{m}{(1-{q}_{t})}^{k-m-1}\sum _{\theta =1}^{\infty }\,Q(\theta )[1-\sum _{s=0}^{\theta -1}\,(\begin{array}{c}m\\ s\end{array})\,{\lambda }^{s}{(1-\lambda )}^{m-s}].$$

The fixed point of the above equation corresponds to *q*_∞_ starting from the initial value *q*_0_ = *ρ*. In general, we obtain *q*_∞_ by solving iteratively Eq. 2 and obtain *R* by replacing the value obtained for *q*_∞_ in Eq. 1.

We further develop the theory for an Erdös-Rényi (ER) graph with an average degree *z* as a simple example. ER graphs in the limit *N* → ∞ clearly satisfy the locally tree-like structure, and hence our theoretical calculation gives a good approximation. Using the degree distribution *P*(*k*) = *e*^−*z*^*z*^*k*^/*k*!, the final fraction of adopted nodes *R* becomes the same as *q*_∞_ since Eqs 1 and 2 become equivalent. Then, the self-consistency equation is simply expressed as

3 $$\begin{array}{rcl}R & = & \rho +(1-\rho )\sum _{\theta =1}^{\infty }\,Q(\theta )[1-{e}^{-z\lambda R}\sum _{i=1}^{\theta }\,\frac{{(z\lambda R)}^{i-1}}{(i-1)!}]\\  & = & \rho +(1-\rho )\sum _{\theta =1}^{\infty }\,Q(\theta )[1-\frac{{\rm{\Gamma }}(\theta ,z\lambda R)}{{\rm{\Gamma }}(\theta )}],\end{array}$$

where Γ(*x*) is the gamma function and Γ(*x*, *y*) is the incomplete gamma function. Thus, for ER networks, we can obtain the fixed point of *R* directly by solving the above self-consistency equation.

## Results

### Phase diagram

For the sake of simplicity, we consider a model with a bimodal distribution of the adoptability *Q*(*θ*) = (1 − *p*)*δ*_*θ*,1_ + *pδ*_*θ*,*n*_ on ER networks. In this setting, a fraction (1 − *p*) of nodes follows simple contagion with *θ* = 1 (simple nodes) and a fraction *p* of nodes follows complex contagion requiring *n* successful exposures to be adopted (complex nodes). We then have three parameters, *p*, *n*, and *λ* which respectively correspond to the fraction of complex nodes, the number of successful exposures required for complex nodes to adopt, and the probability of transmission. Further assuming that the initial density of seed nodes is negligible, i.e, *ρ* → 0, the self-consistency equation becomes,

4 $$R=(1-p)(1-{e}^{-z\lambda R})+p[1-\frac{{\rm{\Gamma }}(n,z\lambda R)}{{\rm{\Gamma }}(n)}].$$

The first term corresponds to the contribution of simple nodes and the second term corresponds to that of complex nodes.

In order to identify a fixed point of Eq. 4, we define $f(R)=-\,R+(1-p)(1-{e}^{-z\lambda R})+p[1-\frac{{\rm{\Gamma }}(n,z\lambda R)}{{\rm{\Gamma }}(n)}]$. Then, the fixed points R^*^ are given by the zeros of *f*(R^*^) = 0. We find that the trivial solution R^*^ = 0 indicates adoption free phase where adoption does not happen both for simple and complex nodes. Adoption phase showing non-zero density of adopted nodes (*R* > 0) appears at the point where the trivial solution R^*^ = 0 becomes unstable. Linear stability analysis implies that the adoption free phase is stable when *f* ′(0) < 0 while it becomes unstable if *f* ′(0) > 0. Thus, the transition between the adoption free phase (*R* = 0) and the adoption phase (*R* > 0) occurs at *f* ′(0) = 0 where $f^{\prime} (R)=-\,1+(1-p)(z\lambda ){e}^{-z\lambda R}+p\frac{{(z\lambda )}^{n}{R}^{n-1}}{{\rm{\Gamma }}(n)}\,{e}^{-z\lambda R}$. From the condition *f* ′(0) = 0, we obtain the transition point *λ*_1_ for any positive integer *n*,

5 $${\lambda }_{1}^{(n,p)}=(\begin{array}{ll}\,\frac{1}{z} & {\rm{if}}\,n=1,\\ \,\frac{1}{z(1-p)} & {\rm{if}}\,n > 1.\end{array}$$

when *n* = 1, all nodes are simple nodes meaning that the model returns to an ordinary simple contagion, i.e., essentially the same as the susceptible-infected-recovered model^13^. Therefore, the threshold for simple contagion model 1/*z* is recovered^15^. When *n* > 1, we get an additional (1 − *p*) factor which corresponds to the fraction of simple nodes.

The nature of the transition at *λ*_1_ is determined by the second derivative of *f*(*R*). While the transition is continuous if *f* ″(0) < 0, it becomes discontinuous if *f* ″(0) > 0. Applying this condition to $f^{\prime\prime} (R)=-\,(1-p)$ ${(z\lambda )}^{2}{e}^{-z\lambda R}-p(1-n+z\lambda R)\frac{{(z\lambda )}^{n}{R}^{n-2}}{{\rm{\Gamma }}(n)}{e}^{-z\lambda R}$, we find that *f* ″(0) < 0 for all values of *p* if *n* > 2. Therefore the transition at *λ*_1_ is always continuous if *n* > 2. However, when *n* = 2, *f* ″(0) < 0 for *p* < 0.5 and *f* ″(0) > 0 for *p* > 0.5, so that the transition is continuous if simple nodes hold a majority (*p* < 0.5) and discontinuous if complex nodes hold a majority (*p* > 0.5). In this case of *n* = 2, we can further identify a tricritical point (*λ*_*tc*_, *p*_*tc*_) = (2/*z*, 1/2) by imposing the condition *f* ″(0) = *f* ′(0) = *f*(0) = 0 where the continuous and discontinuous transition lines meet. At the tricritical point, the size of the discontinuous jump for *p* < 0.5 reduces to zero.

**Figure 2:**
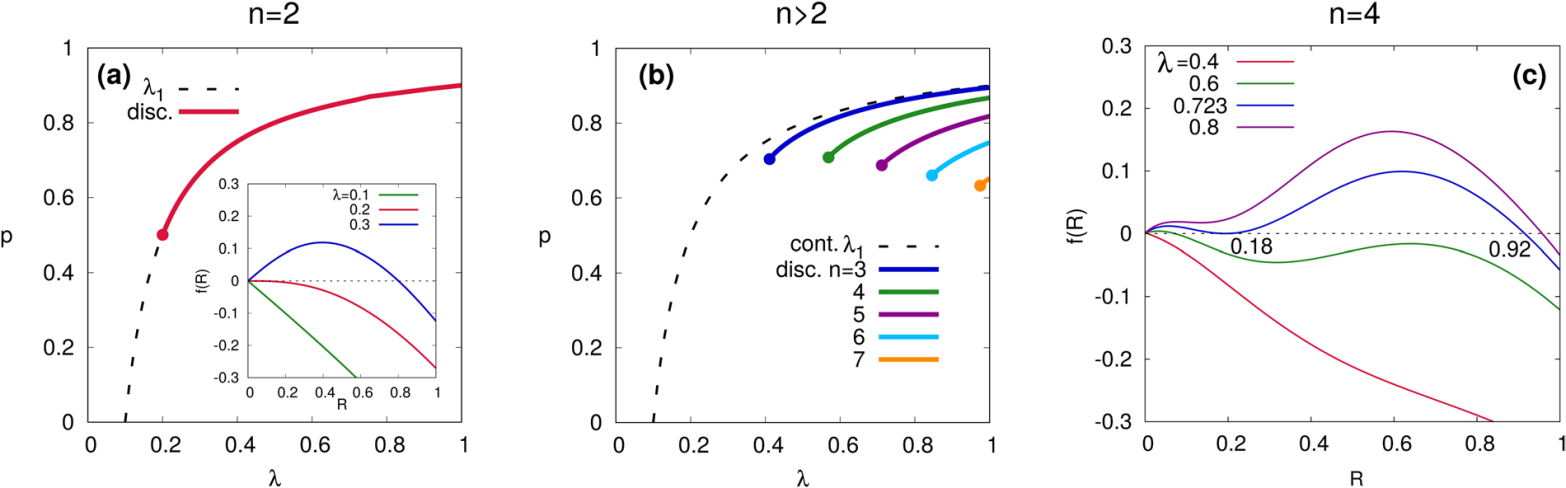
Phase diagram of a generalized contagion model with (**a**) *n* = 2 and (**b**) *n* > 2 for ER networks with *z* = 10. Continuous and discontinuous transition lines are respectively indicated as dashed and solid lines, and (tri)critical points are indicated by filled circles. Graphical solution of *f* (*R*) at *p* = *p*_*tc*_ = 1/2 and *λ* = 0.1, 0.2(*λ*_*tc*_), 0.3 is shown in the inset of (**a**). (**c**) Graphical solution of *f* (*R*) with *n* = 4, *p* = 0.8 and *λ* = 0.4, 0.6, 0.723(*λ*_2_), 0.8 is shown.

In the phase diagram with *n* = 2 for ER networks with *z* = 10 [Fig. 2(a)], we find continuous (dashed) and discontinuous (solid) transition lines and a tricritical point (*λ*_*tc*_, *p*_*tc*_) = (0.2, 0.5) at which the two lines meet [Fig. 2(a)]. For *p* < *p*_*tc*_, the transition at *λ*_1_ is continuous with the scaling behavior $R\sim {(\lambda -{\lambda }_{1})}^{{\beta }_{1}}$ and the exponent *β*_1_ = 1 (derivation in Supplementary Information), the same as the mean-field exponent of an ordinary bond percolation^15^. Approaching the tricritical point, we obtain a different scaling $R\sim {(\lambda -{\lambda }_{tc})}^{{\beta }_{tc}}$ with *β*_*tc*_ = 1/2 (derivation in Supplementary Information). For *p* > *p*_*tc*_, the transition at *λ*_1_ becomes discontinuous. In the inset of Fig. 2(a), the graphical solution *f*(*R*) with respect to *R* at *p* = *p*_*tc*_ with *λ* = 0.1, 0.2, 0.3 is shown. The zeroes of *f*(*R*) correspond to the fixed point and *λ* = 0.2 corresponds to the tricritical point *λ*_*tc*_ in our example with *z* = 10. In the adoption free phase, there exists only a trivial solution which is R^*^ = 0. When *λ* is larger than the tricritical point value (*λ* > *λ*_*tc*_), a new stable solution appears at a non-zero value of R^*^ and R^*^ = 0 solution becomes unstable.

For *n* > 2, in addition to the continuous transition at *λ*_1_ with the critical exponent *β*_1_ = 1 for all *n* > 2, there is another transition at ${\lambda }_{2}^{(n,p)}$ which is discontinuous, indicated by a solid line [Fig. 2(b)]. It is worthwhile to note that *λ*_2_ is larger than *λ*_1_ for any *n* > 2. The location of *λ*_2_ can be analytically identified from the condition *f* ′(R^*^) = 0 with R^*^ ≠ 0. When *n* > 2, the continuous transition line *λ*_1_ and the discontinuous line *λ*_2_ are separated and do not meet. Thus, the tricriticality at which the continuous and discontinuous transition lines meet is a peculiar behavior only found in *n* = 2. The size of the discontinuous jump at *λ*_2_ decreases with decreasing *p* and goes to zero at a critical point (*λ*_*c*_, *p*_*c*_) indicated by a filled circle, at which *f* ″(R^*^) = *f* ′(R^*^) = 0. Thus, the discontinuous transition line ends at the critical point and there is no the second phase transition when *p* < *p*_*c*_. In this regime (*p* < *p*_*c*_), *R* increases gradually without discontinuity when increasing *λ* with *λ* > *λ*_1_. In addition, the discontinuous jump and critical point can disappear as *n* increases for a given *z*, i.e, for *z* = 10, there is no second transition when *n* > 7.

When *p* > *p*_*c*_(*n*) with *n* > 2, the adoption phase is separated into two distinct phases by a boundary at *λ*_2_: simple adoption (low *R*) and complex adoption (high *R*) phases. In addition, the transition at *λ*_2_ has hybrid characteristics showing both discontinuity and a scaling behavior, $R({\lambda }_{2})-R\sim {({\lambda }_{2}-\lambda )}^{{\beta }_{2}^{(n > 2)}}$ with the exponent ${\beta }_{2}^{(n > 2)}=\mathrm{1/2}$ for any *n* > 2 (derivation in Supplementary Information). In addition, when *λ* approaches the critical point a cube-root scaling appears as $R-R({\lambda }_{c})\sim {(\lambda -{\lambda }_{c})}^{{\beta }_{c}}$ where *β*_*c*_ = 1/3 (derivation in Supplementary Information). This is the same scaling found in heterogeneous *k*-core percolation^46^. Such hybrid phase transition also known as mixed phase transition has been observed widely in cooperative percolation in networks such as *k*-core percolation^46,50,51^, bootstrap percolation^48^, percolation of interdependent networks^52–54^, and cooperative epidemic processes^22–25^. A hybrid transition is also predicted in a model of spin chains with long-range interactions^55^, DNA denaturation^56^, and jamming^57,58^, and recently observed experimentally in a colloidal crystal^59^.

An example of how to identify a phase transition is shown in terms of the graphical solution of *f*(*R*) with *z* = 10 and *n* = 4 in the limit *ρ* → 0 [Fig. 2(c)]. The zeroes of *f*(*R*) give the fixed point values of *R* and their stability is given by the derivative of *f*(*R*). First, *R* remains zero for *λ* < *λ*_1_. When *λ*_1_ < *λ* < *λ*_2_, *R* increases gradually as *λ* increases until the second transition at *λ* = *λ*_2_. As *λ* increases further *λ* > *λ*_2_, a complex adoption phase (*R* ≈ 0.92) appears suddenly from the simple adoption phase (*R* ≈ 0.18). Therefore, our analysis predicts the emergence of a double transition showing a continuous and a subsequent discontinuous transition.

### Continuous, discontinuous, and double phase transitions

**Figure 3:**
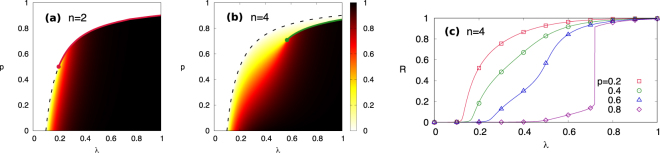
Phase diagram of a generalized contagion model with (**a**) *n* = 2 and (**b**) *n* = 4 showing the final fraction of adopted nodes *R*. Continuous and discontinuous transition lines are respectively indicated as dashed and solid lines, and (tri)critical points are indicated by filled circles. (**c**) The final fraction of adopted nodes *R* vs. *λ* with *n* = 4 for ER networks with *N* = 10^5^ and *z* = 10, averaged over 10^4^ independent runs. Numerical simulations (symbol) and theoretical calculation (line) are shown together. Error bars are smaller than symbols.

We examine the phase diagram and the fraction of adopted nodes for two specific scenarios where *n* = 2 [Fig. 3(a)] and *n* = 4 [Fig. 3(b)] on ER networks with *z* = 10. For *n* = 2, when *p* < 0.5 a typical continuous phase transition occurs at *λ*_1_ [Fig. 3(a)]. But, when more than half of the nodes follow complex contagion (*p* > 0.5), the transition between the adoption free phase and the adoption phase becomes discontinuous. Such discontinuity disappears at a tricritical point at which (*λ*_*tc*_, *p*_*tc*_) = (0.2, 0.5) with *z* = 10. Therefore, for *n* = 2 and varying *λ* there is a single transition at *λ*_1_ either continuous for *p* < *p*_*tc*_ or discontinuous for *p* > *p*_*tc*_.

However, for *n* = 4, we find that the continuous and discontinuous transition lines are separated [Fig. 3(b)]. To be specific, at a given *p* > *p*_*c*_ the location of the discontinuous transition *λ*_2_ appears at a value *λ* > *λ*_1_. The size of the jump decreases with decreasing *p* and the jump disappears at a critical point (*λ*_*c*_, *p*_*c*_) = (0.59, 0.71). Therefore above the critical point (*p* > *p*_*c*_), the size of adopted nodes *R* abruptly changes from simple adoption phase (low *R*) to complex adoption phase (high *R*) at *λ* = *λ*_2_. In contrast, below the critical point (*p* < *p*_*c*_), *R* changes gradually without discontinuity, so that a sharp distinction between simple adoption phase and complex adoption phase does no longer exist.

The continuous and discontinuous transitions with *n* = 4 and *z* = 10 for *p* = 0.2, 0.4, 0.6, and 0.8 are shown in Fig. 3(c). We first note that the theory (line) and numerical simulations (symbol) of *R* for ER networks with *N* = 10^5^ and 100 seed nodes show perfect agreement. In addition, the stark difference between a discontinuous jump for *p* > *p*_*c*_ and a gradual increase of *R* for *p* < *p*_*c*_ is highlighted. Note that the fraction of initial seed cannot be negligible in the finite size networks simulated while it becomes asymptotically small in the thermodynamic limit *N* → ∞.

Moreover, when *p* > *p*_*c*_, for instance *p* = 0.8, the system undergoes a double phase transition with increasing *λ*: a continuous transition from adoption free phase to simple adoption phase, followed by a following discontinuous transition between the simple adoption phase and a complex adoption phase. Recently, multiple transitions in a percolation-type process have been observed in complicatedly designed networks such as clustered networks^60^ and interdependent networks^61^, or with nontrivial percolation protocols such as explosive percolation^62,63^ and asymmetric percolation^64^. In this study, however we find a double transition on simple random networks as a result of competing contagion processes. It is worthwhile to note that in the limiting case *λ* = 1 our model shares a similarity to the heterogeneous *k*-core percolation which also shows a multiple transition^46^.

### Mechanism of double phase transition and mixed phase

**Figure 4:**
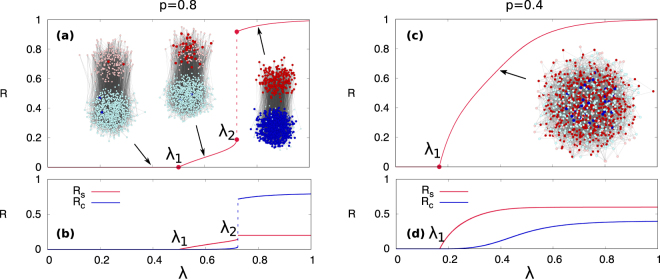
(**a**) Final fraction of adopted nodes *R* as a function of *λ* is shown for ER networks with *N* = 10^5^, *z* = 10, *n* = 4, and *p* = 0.8 (*p* > *p*_*c*_). Network examples are obtained with the same parameters but for a small network *N* = 10^3^ for illustration. Susceptible nodes with simple and complex contagion are indicated by light red and light blue symbols, respectively. Adopted nodes with simple and complex contagion are represented as dark red and dark blue, respectively. (**b**) Final fraction of adopted nodes with simple contagion *R*_*s*_ and complex contagion *R*_*c*_ is shown. (**c**) *R* and (**d**) *R*_*s*_ and *R*_*c*_ are shown for *p* = 0.4 (*p* < *p*_*c*_), disappearing the distinction between simple adoption phase and complex adoption phase.

The underlying mechanism of the double phase transition is illustrated in Fig. 4(a), for ER networks with *z* = 10, *n* = 4, and *p* = 0.8. In a adoption free phase (*λ* < *λ*_1_), most nodes, regardless of being simple or complex contagion nodes, remain susceptible except initial seeds. At *λ*_1_, simple nodes start to become adopted continuously and the system turns into the simple adoption phase (low *R*). As increasing *λ* above *λ*_1_, more and more simple nodes become adopted. But, complex nodes still remain susceptible until *λ* reaches the second transition *λ* = *λ*_2_. Therefore, in the simple adoption phase (*λ*_1_ < *λ* < *λ*_2_) simple contagion nodes are adopted while most of the complex contagion nodes are still susceptible. As *λ* increases further, at the second transition *λ* = *λ*_2_ a bunch of nodes with either simple or complex contagion become adopted abruptly. Thus, in the complex adoption phase (*λ* > *λ*_2_) most nodes are adopted, leading to high *R*. Our numerical simulations for the behavior of the susceptibility of *R* in the limit *ρ* → 0 are compatible with a double transition (see Supplementary Information).

The final fraction of adopted nodes with simple contagion *R*_*s*_ and complex contagion *R*_*c*_ clearly shows the difference between the simple adoption phase and the complex adoption phase as well as the different mechanisms leading to these two transitions [Fig. 4(b)]. In the simple adoption phase, some of simple nodes become adopted but complex nodes remain susceptible so that *R*_*c*_ remains zero and *R*_*s*_ has a finite value. However, in the complex adoption phase, both types of nodes are adopted, so that both *R*_*s*_ and *R*_*c*_ show a high value after a discontinuous jump at *λ*_2_. Note that the maximum of *R*_*s*_ is 0.2 and that of *R*_*c*_ is 0.8 because *p* = 0.8 in this example.

When *p* < *p*_*c*_, the discontinuous transition disappears and a single continuous transition exists at *λ*_1_ [Fig. 4(c)]. As an example, for *p* = 0.4 which is less than *p*_*c*_ = 0.71 both simple nodes and complex nodes start to be adopted at *λ*_1_. And the fraction of adopted nodes with simple *R*_*s*_ and complex *R*_*c*_ adoption gradually increases [Fig. 4(d)]. In the network illustration for *λ* = 0.4 [Fig. 4(c)], we can observe simultaneously simple nodes and complex nodes that are in the adopted state. In this mixed phase, simple and complex nodes are strongly interrelated and a sharp distinction between a simple and a complex adoption phase is no longer possible.

## Discussion

In this study, we have proposed a generalized model of contagion processes unifying simple and complex contagion by introducing an heterogeneous adoptability *Q*(*θ*) together with a transmission probability, or link activation probability, that by a simple contagion mechanism triggers a cascading complex contagion. Our model gives rise to diverse phase transitions such as a continuous transition from adoption free phase to adoption phase, a discontinuous (hybrid) transition between low adoption and high adoption phase, tricriticality at which two lines of the continuous and discontinuous transitions meet, criticality where the discontinuous transition disappears, and a double transition showing successive occurrence of continuous and discontinuous phase transitions when varying the transmission probability *λ*. Specifically, when *n* = 2 a continuous transition becomes discontinuous at a tricritical point. In addition, when *n* > 2 continuous and discontinuous transition lines are separated and two transitions can happen sequentially with increasing *λ*, leading to a double transition. Our model provides a direction to study general contagion processes and shows that heterogeneity in agents’ response to adoption alters significantly the consequences of contagion processes. Further studies may be needed to confirm the finite size effect of the fraction of seed nodes, the effect of heterogeneity in network topology, and more general adoptability distributions, to name a few.

## Electronic supplementary material

Supplementary Information
