# Supplementary material for: Competing contagion processes: Complex contagion triggered by simple contagion
Source: Sci Rep. 2018 Jul 10;8:10422. doi: 10.1038/s41598-018-28615-3 (PMC6039514; doi:10.1038/s41598-018-28615-3)
Supplement: Supplementary file 1 — Supplementary Information [file 41598_2018_28615_MOESM1_ESM.pdf]

Supplementary Information

Competing contagion processes: Complex contagion triggered by simple contagion

Byungjoon Min[1,2*] and Maxi San Miguel[1,†]

[1]IFISC, Instituto de Física Interdisciplinar y Sistemas Complejos (CSIC-UIB), Campus Universitat Illes Balears, E-07122 Palma de Mallorca, Spain
[2]Department of Physics, Chungbuk National University, Cheongju, Chungbuk 28644, Korea
[*]byungjoon@ifisc.uib-csic.es
[†]maxi@ifisc.uib-csic.es

ABSTRACT

In this Supplementary Material, we present the derivation of analytical equations, the derivation of critical exponents, and the susceptibility of a double phase transition.

Derivation of analytical approach with heterogeneous adoptability

Our goal of this calculation is obtaining the fraction of adopted nodes at a steady state. We first assume that a network has a locally tree-like structure in the limit $N \to \infty$. We consider a tree structure with a degree distribution $P(k)$ by replacing a random graph. At the top level ($t \to \infty$), a node randomly chosen has k neighbors drawn from a degree distribution $P(k)$. Each of its neighbor, say node i, in the lower level has $k_i - 1$ accessible neighbors excluding a connected node in the top level where k_i is the degree of a node i. Then, each node j in the subsequent level has $k_j - 1$ neighbors in the next lower level.

We then define q_t as the probability that a node at level t is adopted which is determined by the state of nodes in the lower level. The probability q_t is composed of two independent cases: i) it is initially adopted as a part of seed nodes with probability ρ or ii) it was initially susceptible with probability $1-\rho$ but will become adopted due to sufficiently many of successful exposures. To be specific, if the number s of successful exposures is the same or larger than its adoptability θ, the susceptible node becomes adopted. In our contagion model, we assume heterogeneous adoptability of each node θ drawn from an adoptability distribution $Q(\theta)$. Then, the probability q_{t+1} with a degree distribution $P(k)$ and an adoptability distribution $Q(\theta)$ is given by[1–3]

$$q_{t+1} = \rho + (1-\rho) \sum_{k=1}^{\infty} \frac{kP(k)}{\langle k \rangle} \sum_{m=1}^{k-1} \binom{k-1}{m} q_t^m (1-q_t)^{k-m-1} \sum_{\theta=1}^{\infty} Q(\theta) F(s,\theta). \tag{1}$$

Here, the first term corresponds to the probability that a randomly chosen node is a part of seeds and the second term represents that a node was not a seed but becomes adopted. The term $\binom{k-1}{m} q_t^m (1-q_t)^{k-m-1}$ gives the probability of choosing m adopted neighbors out of total $k-1$ neighbors, with the probability q_t being adopted. In addition, $F(s,\theta)$ represents the response function of the chance that a susceptible node with s exposures and θ adoptability becomes adopted, which is given by

$$F(s,\theta) = \begin{cases} 0 & \text{if} \quad s < \theta \\ 1 & \text{if} \quad s \geq \theta \end{cases} \tag{2}$$

Note that each trial of exposures by adopted neighbors is independent and each adopted neighbor has only one chance at transmission to spread. Then, the probability of s exposures out of every trials by m adopted neighbors with a probability λ can be expressed as $\binom{m}{s} \lambda^s (1-\lambda)^{m-s}$. Combining all these factors, we can derive the recursive

equation for q_{t+1},

$$q_{t+1} = \rho + (1-\rho) \sum_{k=1}^{\infty} \frac{kP(k)}{\langle k \rangle} \sum_{m=1}^{k-1} \binom{k-1}{m} q_t^m (1-q_t)^{k-m-1} \sum_{\theta=1}^{\infty} Q(\theta) \left[1 - \sum_{s=0}^{\theta-1} \binom{m}{s} \lambda^s (1-\lambda)^{m-s} \right]. \tag{3}$$

The fixed point q_∞ can be calculated by solving the recursive equation starting from the initial value $q_0 = \rho$.

After calculating q_∞, the expected final fraction of adopted nodes R from a fraction ρ of seed nodes chosen randomly can be expressed as

$$R = \rho + (1-\rho) \sum_{k=0}^{\infty} P(k) \sum_{m=0}^{k} \binom{k}{m} q_\infty^m (1-q_\infty)^{k-m} \sum_{\theta=1}^{\infty} Q(\theta) \left[1 - \sum_{s=0}^{\theta-1} \binom{m}{s} \lambda^s (1-\lambda)^{m-s} \right]. \tag{4}$$

The term $\binom{k}{m} q_\infty^m (1-q_\infty)^{k-m}$ corresponds to the probability of having m adopted neighbors out of k neighbors. And, the term $\left[1 - \sum_{s=0}^{\theta-1} \binom{m}{s} \lambda^s (1-\lambda)^{m-s}\right]$ represents the probability that the number of successful exposures with the transmission probability λ from m adopted neighbors is the same or larger than the adoptability θ. In summary, we first obtain q_∞ by solving iteratively Eq. 3 and obtain R by putting q_∞ into Eq. 4.

Derivation of critical exponents

The self-consistency equation of R for ER networks is given by

$$R = (1-p)\left(1 - e^{-z\lambda R}\right) + p\left[1 - \frac{\Gamma(n, z\lambda R)}{\Gamma(n)}\right], \tag{5}$$

where R is the the final fraction of adopted nodes, p is the fraction of complex nodes, z is the mean degree, λ is the probability to transmission, and n is the number of successful exposures for complex nodes. For further analysis, we define

$$f(R,\lambda) = -R + (1-p)\left(1 - e^{-z\lambda R}\right) + p\left[1 - \frac{\Gamma(n, z\lambda R)}{\Gamma(n)}\right]. \tag{6}$$

Then, the zeros of Eq. 6 gives the fixed points R^*.

We then analyze a critical exponent around phase transition. First, we analyze critical exponents around $\lambda_1 = 1/z(1-p)$ for ER networks. We can expand $f(R,\lambda)$ near $\lambda \approx \lambda_1$ and $R \approx 0$ as

$$-R + (1-p)z\lambda R - \frac{1}{2}(1-p)(z\lambda R)^2 + \cdots = 0 \tag{7}$$

For $\lambda \approx \lambda_1$ where $\lambda_1 = 1/z(1-p)$, R shows the relation,

$$R \sim (\lambda - \lambda_1)^{\beta_1}, \quad \beta_1 = 1. \tag{8}$$

We next check the critical exponent near tricritical point at $p_{tc} = 1/2$ for $n = 2$. Eq. 6 with $p = 1/2$ and $n = 2$ becomes

$$f(R,\lambda) = -R + \frac{1}{2}\left[1 - e^{-z\lambda R}\right] + \frac{1}{2}\left[1 - e^{-z\lambda R}(1 + z\lambda R)\right]. \tag{9}$$

Expanding around $R \approx 0$, we obtain the relation

$$-2R + z\lambda R - \frac{1}{6}(z\lambda R)^3 + \cdots = 0. \tag{10}$$

Rearranging the equation, the final fraction of adopted node around the tricritical point $\lambda_{tc} = 2/z$ satisfies

$$R \sim (\lambda - \lambda_{tc})^{\beta_{tc}}, \quad \beta_{tc} = 1/2, \tag{11}$$

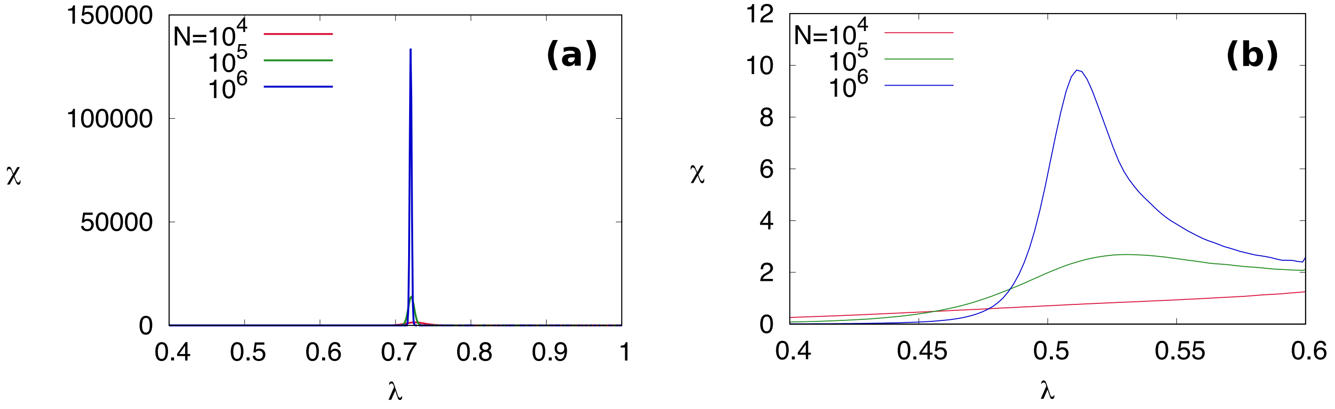

Figure 1. (a) Susceptibility χ of two peaks and (b) zoom-in view of the first peak as a function of λ obtained by numerical simulation with $N = 10^4, 10^5, 10^6$ for ER networks, $n = 4$, and $p = 0.8$ in the limit $\rho \to 0$. The peaks persist as N increases.

for $n = 2$ and $p_{tc} = 1/2$. Next, in order to determine the critical exponent in hybrid (mixed) phase transitions, we expand $f(R,\lambda)$ near R_2 and λ_2.

$$\delta f(R_2,\lambda_2) = \left.\frac{\partial f}{\partial R}\right|_{R_2,\lambda_2} \delta R + \left.\frac{\partial f}{\partial \lambda}\right|_{R_2,\lambda_2} \delta\lambda + \frac{1}{2}\left.\frac{\partial^2 f}{\partial R^2}\right|_{R_2,\lambda_2} (\delta R)^2 + \frac{1}{2}\left.\frac{\partial^2 f}{\partial \lambda^2}\right|_{R_2,\lambda_2} (\delta\lambda)^2 + \frac{1}{2}\left.\frac{\partial^2 f}{\partial R \partial \lambda}\right|_{R_2,\lambda_2} (\delta R)(\delta\lambda) + \cdots \tag{12}$$

At a line of discontinuous jump, $f(R_2,\lambda_2) = \left.\frac{df}{dR}\right|_{R_2,\lambda_2} = 0$. Therefore, the lowest terms, $(\delta R)^2$ and $\delta\lambda$ which have the opposite sign each other, shows the relation $\delta R \sim (\delta\lambda)^{\beta_2}$ and $\beta_2 = 1/2$ with $\delta\lambda \ll 1$. Finally, we obtain the critical exponent near critical point. Since $f(R_c,\lambda_c) = \left.\frac{df}{dR}\right|_{R_c,\lambda_c} = \left.\frac{d^2 f}{dR^2}\right|_{R_c,\lambda_c} = 0$ for the critical point, $\delta R \sim (\delta\lambda)^{\beta_c}$ and $\beta_c = 1/3$ with $\delta\lambda \ll 1$ regardless of n if $n \geq 3$. Note that the discontinuous jump and critical point can disappear as n increases for a given z, i.e, for $z = 10$, there is no second transition when $n > 7$.

Susceptibility of double phase transitions

In order to confirm a double phase transition, we numerically obtain a susceptibility as a function of λ for different $N = 10^4, 10^5, 10^6$ in the limit $\rho \to 0$. We do that by keeping a constant number of seed nodes $n_i = 100$ for different values of N so that $\rho = n_i/N \to 0$ as $N \to \infty$. We define a standard susceptibility as[4]

$$\chi = N(\langle R^2\rangle - \langle R\rangle^2), \tag{13}$$

In a finite system, however the fraction of seed nodes ρ cannot be vanishing since it becomes at least $1/N$ for a single seed. But a finite ρ in cooperative spreading processes can effect significantly phase transition and critical phenomena.[5,6] In this section, we focus on the susceptibility (the fluctuation of order parameter) in the limit $\rho \to 0$ and $N \to \infty$ in order to check numerically the double transition that our theory predicts.

We find that two peaks in the susceptibility are located at the same position as our theory predicts. In addition, the two peaks persist as N increases and thus are compatible with the double phase transition. By checking the susceptibility for ER networks with different N (Fig. 1), we confirm numerically that the double phase transition is genuine in the limit $\rho \to 0$. For finite ρ, a numerical study of the finite size scaling would be needed in order to address the effect of the fraction of initial seeds in our model.

References

1. J. P. Gleeson and D. J. Cahalane, Seed size strongly affects cascades on random networks, Phys. Rev. E **75**, 056103 (2007).
2. C. D. Brummitt, K.-M. Lee, and K.-I. Goh, Multiplexity-facilitated cascades in networks, Phys. Rev. E **85**, 045102(R) (2012).
3. M. E. J. Newman, Spread of epidemic disease on networks, Phys. Rev. E **66**, 016128 (2002).
4. P. Colomer-de-Simón and M. Boguñá, Double percolation phase transition in clustered complex networks, Phys. Rev. X **4**, 041020 (2014).
5. Choi, W., Lee, D. & Kahng, B. Mixed-order phase transition in a two-step contagion model with a single infectious seed. Phys. Rev. E **95**, 022304 (2017).
6. Choi, W., Lee, D. & B. Kahng, Critical behavior of a two-step contagion model with multiple seeds. Phys. Rev. E **95**, 062115 (2017).
